# 

**Published:** 1884-07

**Authors:** 


					﻿J ULY>884.
XQLoJsEFlIES
Wu.tI.-XXV
NEW SEklEs*!
vol. i f-Nj>.L5-ti II E Z
& 1855 O

IIedw^W'
» Journa^B
kVrt
sZpby* WJ.WESTMOREtANfllK
...Wk H.V.M.MILLER,M.D.LL.D.>/I
ZW	JAMES A.GRAY, M.D. ?«k
d/lBLlSHED BY JAMES P.HARRISON&COlW
Jlu	tflTLANTA.GA.
SUBSCRIPTION : 2.50 A YEAR.
				

